# Influence of magnetic nanoparticle biotransformation on contrasting efficiency and iron metabolism

**DOI:** 10.1186/s12951-022-01742-w

**Published:** 2022-12-17

**Authors:** Alexey V. Yaremenko, Ivan V. Zelepukin, Ilya N. Ivanov, Roman O. Melikov, Nadezhda A. Pechnikova, Dzhuliia Sh. Dzhalilova, Aziz B. Mirkasymov, Vera A. Bragina, Maxim P. Nikitin, Sergey M. Deyev, Petr I. Nikitin

**Affiliations:** 1 Center for Nanomedicine, Brigham and Women’s Hospital, Harvard Medical School, Boston, MA 02115 USA; 2 Shemyakin-Ovchinnikov Institute of Bioorganic Chemistry of the Russian Academy of Sciences, 117997 Moscow, Russia; 3 School of Medicine, Aristotle University of Thessaloniki, 54124 Thessaloniki, Greece; 4 National Research Nuclear University MEPhI (Moscow Engineering Physics Institute), 115409 Moscow, Russia; 5 Pirogov Russian National Research Medical University, 117997 Moscow, Russia; 6 Engelhardt Institute of Molecular Biology of Russian Academy of Sciences, 119991 Moscow, Russia; 7 Saint Petersburg State University, 199034 Saint Petersburg, Russia; 8 Saint Petersburg Pasteur Institute, 197101 Saint Petersburg, Russia; 9 Avtsyn Research Institute of Human Morphology of Federal State Budgetary Scientific Institution, Petrovsky National Research Centre of Surgery, 117418 Moscow, Russia; 10 Prokhorov General Physics Institute of the Russian Academy of Sciences, 119991 Moscow, Russia; 11 Sirius University of Science and Technology, 354340 Sirius, Russia; 12 Moscow Center for Advanced Studies, 123592 Moscow, Russia

**Keywords:** Nanoparticle degradation, Mononuclear phagocyte system, Biodistribution, Pharmacokinetics, Toxicity, Magnetic nanoparticles, Iron gene expression

## Abstract

Magnetic nanoparticles are widely used in biomedicine for MRI imaging and anemia treatment. The aging of these nanomaterials in vivo may lead to gradual diminishing of their contrast properties and inducing toxicity. Here, we describe observation of the full lifecycle of 40-nm magnetic particles from their injection to the complete degradation in vivo and associated impact on the organism. We found that in 2 h the nanoparticles were eliminated from the bloodstream, but their initial biodistribution changed over time. In 1 week, a major part of the nanoparticles was transferred to the liver and spleen, where they degraded with a half-life of 21 days. MRI and a magnetic spectral approach revealed preservation of contrast in these organs for more than 1 month. The particle degradation led to the increased number of red blood cells and blood hemoglobin level due to released iron without causing any toxicity in tissues. We also observed an increase in gene expression level of Fe-associated proteins such as transferrin, DMT1, and ferroportin in the liver in response to the iron particle degradation. A deeper understanding of the organism response to the particle degradation can bring new directions to the field of MRI contrast agent design.

**Supplementary Information:**

The online version contains supplementary material available at 10.1186/s12951-022-01742-w.

## Introduction

Magnetic particles (MPs) have great potential in medicine for diagnosis, imaging, and therapy of various diseases. Currently, MPs are already applied in the clinic for the treatment of iron deficiency anemia [1] and undergo clinical trials for the treatment of prostate cancer, osteosarcoma and corneal edema [1]. Besides, a prototype of an anti-COVID-19 vaccine was developed based on MPs [2]. Since the early 1990s, MPs have been used as contrast agents for magnetic resonance imaging to improve the diagnosis of various diseases including lymph node metastases and liver lesions [3, 4]. Likewise, novel types of extra-sensitive tomographic techniques based on the analysis of nonlinear magnetic properties of MPs have been developed and are under investigation now [5–7].

For safe application of nanoparticles (NPs) in vivo, it is important to fully understand their fate in the organism from the moment of their administration to complete decomposition and elimination from the body. Superparamagnetic nanoparticle contrast agents are usually crystals consisting of iron oxides stabilized by various organic polymers or dense inorganic shells. They can be accumulated and stored in the organism for a long time. In previous in vitro studies, it was shown that magnetic nanoparticles bind with cell within several minutes, triggering early endocytosis. This process is accompanied by the change of mechanical properties of the cell [8] and is followed by the transfer of nanoparticles to lysosomes, where they reside for at least one month [9].

The biodegradation process of nanoparticles is governed by the harsh lysosomal environment and enzymatic activity of various biomolecules [10, 11]. Protein adsorption on MPs in serum leads to their opsonization, cellular uptake and partial aggregation in lysosomes. After that, chemical corrosion and enzymatic attack on the MP surface cause degradation of iron oxide crystals inside the cells [12]. The nanoparticle biodegradation can result in oxidative stress due to the release of Fe^3+^ ions, as well as biogenic iron overloading [13, 14]. At the same time, a hyperaccumulation of iron leads to a change of its metabolism, including the expression level of iron-containing and iron transporter proteins such as ferritin, ferroportin, divalent metal transporter 1 (DMT1), hemoglobin, etc. Various strategies were designed to slowdown degradation of magnetic nanoparticles, for example coating of magnetite cores with gold shell, dense polystyrene or with highly chelating polyacrylic acid, as well as clustering of nanoparticles on the cell membrane prior their uptake [9–11].

Understanding the biodegradation rate of magnetic nanoparticles, as well as subsequent increasing of the level of iron-containing metabolites in vivo is important to assess MP diagnostic window in the body. Iron degradation products generally have much less magnetic contrast [12] and can change its position in the body due to iron transport processes [13]. In addition, a possibility of the biosynthesis of magnetite nanoparticles de novo in human cells from the dissolvable iron species was shown [14]. Moreover, the diagnostic capacity of the spectral magnetic techniques and MRI can differ significantly. It was shown previously that iron-containing products can induce long-term MRI contrast even after the full nanoparticle degradation [12].

Usually, the process of nanoparticle elimination from the body is analyzed by measuring concentration of their components using mass spectrometry [15, 16]. However, this method does not allow tracking the processes of the internal transformations of the nanoparticles, for example, changes in their crystal structure and magnetic properties. Sometimes MPs degradation is accompanied by alterations of magnetic susceptibility of the material without any variations of MP concentration or even shape [17]. Besides, iron released during degradation mostly doesn’t leave the organism but transforms into ferritin or hemosiderin. That leads to weakening of magnetic susceptibility of the tissue but doesn’t cause a decrease of iron concentration in the body [18]. Therefore, it seems promising to analyze the magnetic properties of nanoparticles in vivo using magnetic techniques. Various methods based on analysis of AC-Susceptibility, SQUID detectors, and magnetic spectroscopy were previously used to analyze the degradation rate of magnetic particles ex vivo and in vivo [7, 9, 19, 20].

**Scheme 1:**
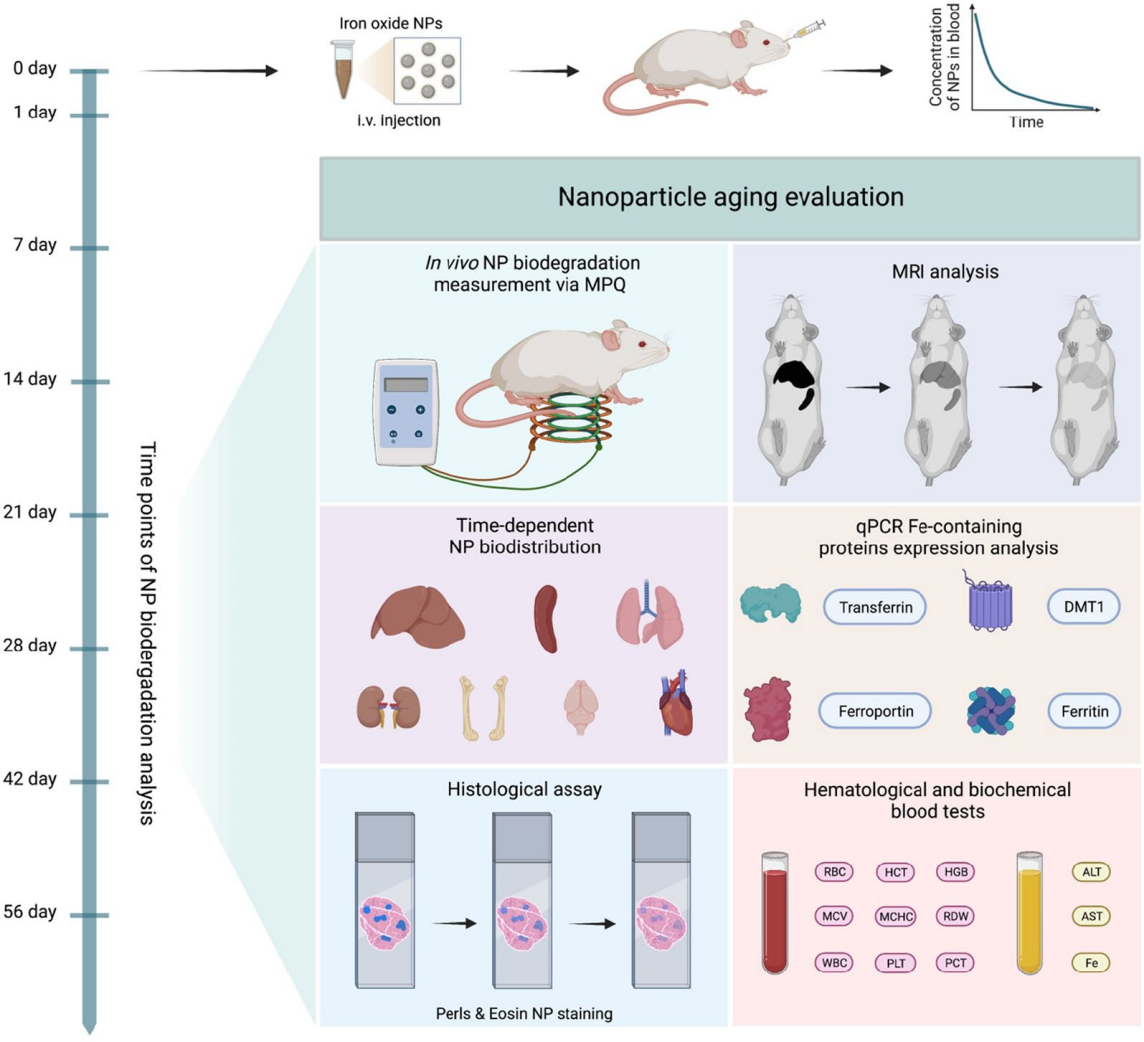
Schematic illustration of investigating magnetic nanoparticles biodegradation

In this work, a complete life cycle of 40-nm magnetic nanoparticles was observed from their administration into the bloodstream to the particle aging in the liver and spleen macrophages, while the toxicity was assessed through the application of several advanced complementary technologies (Scheme 1). We show that magnetite nanoparticles coated with glucuronic acid can remain in the body for several months with the degradation half-life of 21 days. There wasn’t detected any significant toxicity during the degradation process. Interestingly, the iron redistribution was observed between the liver and spleen, and more importantly, between the cells inside the organs. In addition, it was found that the nanoparticle degradation significantly increases the expression of iron-related proteins: DMT1, ferroportin, and transferrin in the liver.

## Materials and methods

### Materials

Magnetic nanoparticles coated with glucuronic acid from Chemicell GmbH (Germany) specified as “50-nm fluidMAG-ARA”; hematoxylin and eosin from Biovitrum (Russia); potassium hexacyanoferrate (II) trihydrate, hydrochloric acid, and formaldehyde from Sigma-Aldrich (USA); K3-EDTA test tubes from Guangzhou Improve Medical Instruments (China); ExtractRNA, CleanRNA Standard kit, MMLV kit, HS Taq DNA Polymerase, and SYBR Green I dye from Evrogen (Russia) were used in the experiments. All other chemicals were of analytical grade.

### Animals

The inbred female BALB/c mice (22–25 g weight) were obtained from Pushchino facility for laboratory animals (Moscow Region, Russia). The animals were stored at the vivarium of Institute of Bioorganic Chemistry (IBCh RAS). All research protocols were approved by the Institutional Animal Care and Use Committee of IBCh RAS. Before the nanoparticle injection, the animals were anesthetized by the mixture of Zoletil (Virbac, France) and Rometar (Bioveta, Czech Republic) at a dosage of active ingredients: Tiletamine-HCl/Zolazepam-HCl/Xylazine-HCl 20/20/1.6 mg/kg. Animal euthanasia was carried out by the cervical dislocation.

### Nanoparticle characterisation

Transmission electron microscopy (TEM) images were obtained with a Zeiss Libra 200 FE (Carl Zeiss Jena Gmbh, Jena, Germany) microscope using an accelerating voltage of 200 kV. Nanoparticles were diluted in distilled water, dropped on the carbon-coated copper grid and dried under air.

Hydrodynamic size and ζ-potential distributions of the nanoparticles were measured using Zetasizer Nano ZS device (Malvern Instruments, U.K.). Distribution by number was used to estimate hydrodynamic size. To analyze colloidal stability of nanoparticles in buffers and serum, distribution by intensity was used, since it less interfered by light scattering with serum proteins. ζ-potentials were measured in 10 mM NaCl.

### Nanoparticle circulation

The in vivo nanoparticle circulation kinetics was measured by the Magnetic Particle Quantification (MPQ) technique [5] as was published earlier [21]. Briefly, a tail of an anesthetized mouse was placed into the MPQ detector measuring coil, and 100 µL of suspension containing 300 µg of magnetic particles in PBS was injected into the retroorbital sinus of the animal. The nanoparticle detection was carried out in the tail vessels of the animals by the MPQ every 2.8 s. The excitation of the magnetic nanoparticles in the coil was performed with an ac magnetic field at frequencies *f*_*1*_ *= 702* Hz, *f*_*2*_ *= 87* kHz with amplitudes *H*_*1*_ *= 64 ± 6* Oe, *H*_*2*_ *= 33 ± 3* Oe, respectively. The induction signal from the nanoparticles was detected at the combinatorial frequency *f*_*2*_ *+ 2f*_*1*_.

### SQUID magnetization analysis

An MPMS XL SQUID magnetometer (Quantum Design, USA) was used for measurements of magnetization curves of samples with and without nanoparticles. For liver tissue analysis, 300 µg of MPs were injected into mouse retroorbital sinus. 24 h after the injection, mice were euthanized, and livers were isolated, weighed, and frozen in liquid nitrogen. Immediately before magnetization measurement, the tissue samples were unfrozen at room temperature and placed for analysis in a plastic tube. The magnetization of the samples was measured against a plastic tube filled with pure water (100 µL). Registration of the magnetization curves and recording of hysteresis loops were carried out in the range of moderate magnetic fields from − 240 to 240 Oe. Next, the magnetization data were normalized to the liver sample mass.

Temperature-dependent magnetization was measured at the field of 50 Oe in 5–250 K temperature range. Zero-field cooled (ZFC) curves were obtained during the heating cycle, while field cooled (FC) curves — during the cooling. Liver samples were placed in plastic tubes and were frozen during the measurements. For nanoparticle measurements, they were diluted in concentration similar to that one in the liver samples and embedded in 1% agarose gel. Nanoparticle concentration was normalized using quantitative MPQ technique.

### MRI imaging

MRI analysis of mice was carried out in an ICON 1T MRI system (Bruker, USA) using a mouse whole-body-volume radio frequency coil. For image acquisition, a two-dimensional spin echo RARE sequence was used with the following parameters: repetition time/echo time (TR/TE) − 3000/8.68 ms; resolution − 300 μm/ pixel; field of view (FOV) − 50 × 80 mm; 15 slices per scan; slice thickness − 1 mm.

### MPQ biodistribution assay

The MPQ technique was used for the nanoparticle biodistribution measurements. Initially, 300 µg of MPs were administered into retroorbital sinus of animals (7 groups, n = 3 mice in each). To measure the MP biodegradation non-invasively, an MPQ detection coil of 20 mm in diameter [7, 9, 22] was attached to the region near the mice liver and spleen, and the magnetic signal was recorded for 10 s.

For ex vivo investigation of nanoparticle degradation, the animals were euthanized 1, 7, 14, 21, 28, 42, and 56 days after the MP injection, and brain, lungs, heart, liver, spleen, kidneys, and bone (femur with bone marrow) were extracted for the MPQ biodistribution analysis. The organs were placed into the MPQ detector coil with a diameter of 20 mm, and the magnetic signal of each organ was normalized to the total signal from all extracted organs of each animal. The data were presented in % of injected dose (% ID).

### Histology

For histological analysis, 1, 7, 14, 28, and 56 days after the MP injection (300 µg in 100 µL of PBS), liver, spleen and kidneys were extracted. The organs were fixed for 1 day in 4% formaldehyde and then embedded in paraffin. Slices of 5 μm thick were made, and then the samples were stained with hematoxylin and eosin for general histological assay, or eosin and Perls Prussian blue for iron visualization. The histomorphology analysis was carried out using the Leica DM2500 microscope with a Leica DFC290 digital camera (Leica Microsystems GmbH, Germany).

### Hematology

The hematological analysis of animals was carried out 1, 7, 14, 28, 42, and 56 days after the MP injection. The blood samples from intact mice without the MP injection were used as a negative control. The blood samples in K3-EDTA test tubes were collected by puncturing the facial vein. A hematology analyzer Zoomed 5180 Vet (URIT Medical Electronic Co Ltd, China) was used to analyze the following blood parameters: red blood cell count (RBC, 10^12^ per L), hematocrit (HCT, %), hemoglobin concentration (HGB, g/dL), mean corpuscular volume (MCV, fL), mean corpuscular hemoglobin (MCH, pg), mean corpuscular hemoglobin concentration (MCHC, g/dL), standard deviation of red cell distribution width (RDW_SD, fL), coefficient of variation of red cell distribution width (RDW_CV, %), white blood cell count (WBC, 10^9^ per L), platelet count (PLT, 10^9^ per L), plateletcrit (PCT, %), platelet-large cell ratio (P_LCR, %).

### Blood biochemistry analysis

The blood biochemistry analysis was carried out 1, 7, 14, 28, and 42 days after the MP injection. The blood samples from intact mice without the MP injection were used as a negative control. The blood samples were collected by puncturing the facial vein. To obtain serum, the blood samples were incubated for 30 min at room temperature, then the samples were centrifuged (15 min, 1800–2200 g), and the serum was kept at − 20 °C until the analysis. A biochemistry Analyzer i-Magic V7 VET (Shenzhen iCubio Biomedical Technology Co, China) was used to analyze the following blood parameters: alanine aminotransferase activity (ALT, IU/L), aspartate aminotransferase activity (AST, IU/L), and iron concentration (Fe, µΜ).

### Real-time PCR for evaluation of Fe-contain protein expression

To study the influence of the magnetic particle degradation on the level of Fe-containing protein gene expression, 12 mice (4 groups of 3 animals in each) were injected with the nanoparticles (300 µg in 100 µL PBS). In 1, 7, 14, and 28 days after the injection, the animals were euthanized, livers were extracted and kept at − 80 °C. The livers from intact mice without the MP injection were used as a negative control. When all samples were collected, total RNA was extracted with an ExtractRNA kit and purified with an CleanRNA Standard kit according to the protocols supplied by the manufacturer. Then, 2 µg of the total RNA were used for reverse transcription with 30 pmol Random (dN)_10_–primer, 30 pmol Oligo(dT)_15_-primer, and 200 U of MMLV reverse transcriptase according to the manufacturer protocols. A real-time PCR (qPCR) was used to determine the expression level of following genes: DMT1, DMT1 (IRE only), Ferritin (heavy and light chains), Ferroportin and Transferrin. The qPCR reactions were carried out in a CFX96 thermal cycler (Bio-Rad, USA) using HS Taq DNA Polymerase, SYBR Green I fluorescent dye, and gene specific primers (Table 1). To obtain a relative expression level of the genes, the results were normalized to the expression level of Glyceraldehyde 3-phosphate dehydrogenase (GAPDH). To avoid getting false or unreliable data, each experiment was triplicated.

**Table 1:** List of gene-specific primers for real-time PCR

	Protein	Forward (5′ → 3′)	Reverse (5′ → 3′)	PCR (bp)
1	DMT1	TCCCACATTCCACTGGAGACT	AGAGCAGCTTAAATCCAGCCA	210
2	DMT1 (IRE only)	CGTCTGCTCCATCAACATGTACT	GTATCTTCGCTCAGCAGGACTTTC	207
3	Ferritin heavy chain	GAAGCTGCAGAACCAGCGAG	CCTGTTCACTCAGATAATACGTCT	215
4	Ferritin light chain	ATCTGCATGCCCTGGGTTC	GAGTGAGGCGCTCAAAGAGA	201
5	Ferroportin	CCGAGATGGATGGGTCTCCT	GCCACATTTTCGACGTAGCC	220
6	Transferrin	GCAGTGTCAGAGCACGAGAA	GGCTTCAGGTTGTTCGGAGT	209
7	GAPDH	TCATGACCACAGTCCATGCC	ATCCACGACGGACACATTGG	212

## Results and discussion

### Nanoparticle characterization

**Fig. 1:**
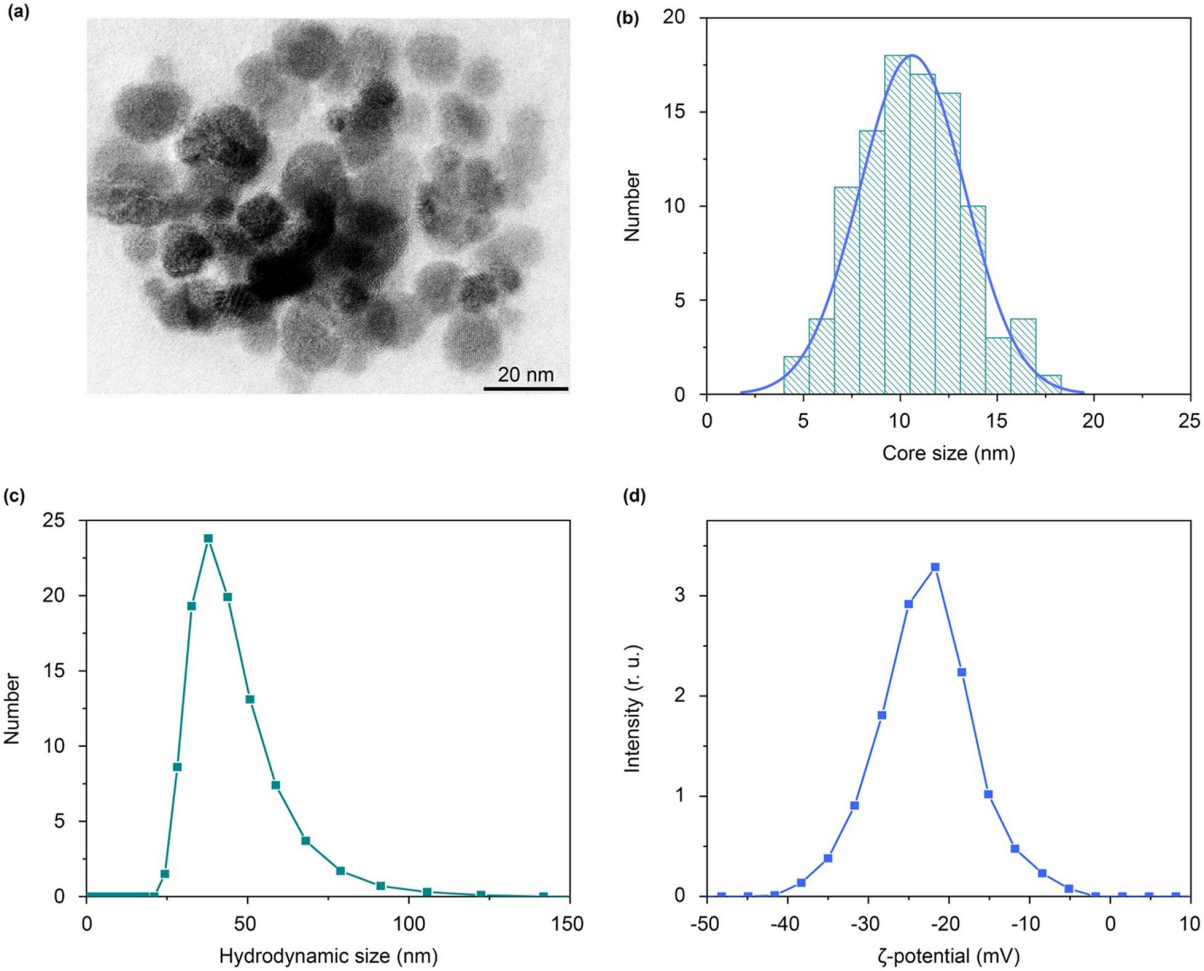
FluidMAG-ARA nanoparticle characterization. **a** TEM image of FluidMAG-ARA nanoparticles. **b** NP size distribution obtained from the TEM images; the blue line shows normal fitting. **c** Distribution of hydrodynamic diameters of nanoparticles. **d** Distribution of particle ζ-potentials

In this study, we investigated the pharmacokinetics of magnetite nanoparticles coated with glucuronic acid polymer FluidMAG-ARA (Chemicell, Germany) with a nominal size of 50 nm. This particle size was selected for our study as an average one for the contrast particles already used in clinic for MRI (20–100-nm agents) [23]. The transmission electron microscopy showed high monodispersity of the magnetite particles with the core size of 11 ± 3 nm (Fig. 1a, b). The hydrodynamic size of the particles in water was 43 ± 10 nm (Fig. 1c, number size distribution), polydispersity index (PDI) was 0.054. This ratio of the physical and hydrodynamic sizes indicated that the FluidMAG-ARA particles are likely multi-core polymer coated particles. The measured particle ζ-potential was − 23 ± 5 mV (Fig. 1d). This value was high enough to provide the NP colloidal stability in serum. The nanoparticles showed no signs of aggregation within 2 h of incubation in water and PBS buffer (Additional file 1: Fig. S1). The hydrodynamic size of the particles in mice serum increased by 10 nm during that time (Additional file 1: Fig. S1), which can be attributed to formation of a protein corona on the NP surface.

### Nanoparticle circulation in blood

**Fig. 2:**
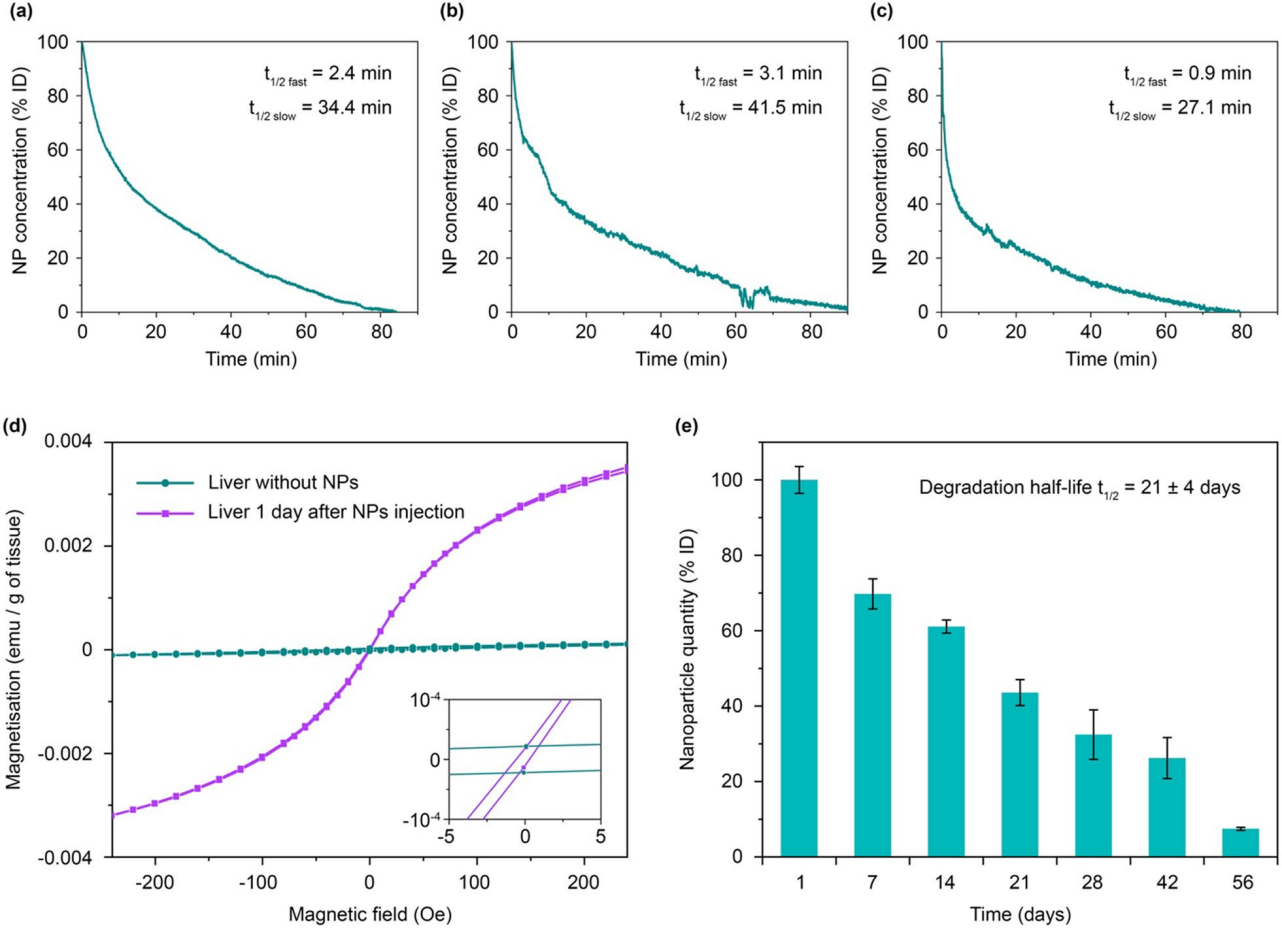
FluidMAG-ARA nanoparticle pharmacokinetics. **a**–**c** Circulation kinetics of FluidMAG-ARA nanoparticles in mice. The curves show kinetics of NP clearance from the bloodstream. **d** SQUID analysis of magnetization of liver tissues before and 1 day after the nanoparticle injection. **e** The kinetics of the NP degradation in vivo measured by MPQ

Then we studied the short-term fate of magnetic nanoparticles in vivo: blood circulation kinetics and initial biodistribution. For the nanoparticle detection, we used the MPQ approach, which was recently updated for biosensing of various analytes using MPs as assay labels [24–26]. The MPQ allows non-invasive measurement of the magnetic particle concentration in murine veins and arteries. Briefly, a mouse tail is placed into the detection coil generating magnetic field at two frequencies: at a low frequency *f*_*1*_ with a high amplitude *H*_*1*_ and at a high frequency *f*_*2*_ with a low amplitude *H*_*2*_. The linear dia- and paramagnetic materials, including iron ions and biological tissues, response only at the applied magnetic field frequencies *f*_*1*_ and *f*_*2*_, while nonlinear ferro- and superparamagnetic materials such as most MPs provide additional responses at combinatorial frequencies of the applied magnetic field *f = nf*_*1*_ *+ mf*_*2*_ (where *n, m* are integers, *n*^*2*^ *+ m*^*2*^ *≠ 0*). This enables extremely sensitive measuring the magnetic particle concentration in biological media. The method also features high time resolution. In this experiment, it was 2.8 s (Fig. 2a–c).

Figure 2a–c shows kinetics of nanoparticle elimination from the bloodstream in 3 different mice. The kinetics were similar in different animals and could be easily fitted by a biexponential function with fast t_1/2 fast_ = 2.2 ± 1.1 min and slow t_1/2 slow_ = 34.4 ± 7.2 min decays. The total time of nanoparticle elimination from the bloodstream was 84 ± 7 min (Fig. 2a–c). The obtained results well correlate with the literature data for non-stealth nanoparticles [21]. Pharmacokinetics in a logarithmic scale (Additional file 1: Fig. S2) showed that in the first 10 min, the particle elimination rate was significantly faster than during the following part of the curve. This can be caused by a saturation of the process of the particle uptake by macrophages due to the slow process of reverse recycling of the membrane receptors of the cells after the endocytosis. This feature was previously discovered for in vivo injections with high doses of nanoparticles [27], as well as during experiments with an ex vivo liver perfusion model [28].

### SQUID magnetization analysis

Next, we used SQUID magnetometry to measure magnetization curves of the liver as the main organ that eliminates MPs from the bloodstream (Fig. 2d). The magnetization curve of a control liver sample without nanoparticles exhibited insignificant nonlinearity and reached saturation at the level of 10^− 4^ emu/g of tissue. Such magnetic behavior of the tissue may be due to the presence of antiferromagnetic ferritin in the liver [29]. After administration of 300 µg of magnetite nanoparticles, the liver magnetization increased 40-fold, and the curve showed superparamagnetic behavior.

Then we analyzed the thermal dependence of magnetization during the field-cooled (FC) and zero-field cooled (ZFC) measurements (Additional file 1: Fig. S3). Blocking temperature (T_B_, roughly the maxima of ZFC curve) shows the transition between the NPs with magnetic moments in a blocked state to particles with a superparamagnetic behavior. In our samples, we observed increase of T_B_ from 163 ± 7 K for FluidMAG-ARA nanoparticles in agarose gel to 190 ± 3 K and 195 ± 7 K for NPs in liver samples 1 and 2 days after injection, respectively. The growth of T_B_ can be corresponding to the increased inter-particle interactions in lysosomes, as was described by Levy et al. [30]. In our study, interaction of magnetic particles increased within first 2 days of accumulation in the liver. Nevertheless, at room temperature nanoparticles retain their superparamagnetic behavior. The significant difference in the sample magnetization while maintaining the nonlinear magnetization-field dependence allowed us to use magnetic spectroscopic techniques such as MPQ to study in vivo and ex vivo the aging processes of FluidMAG-ARA nanoparticles.

### MPQ contrast aging

For non-invasive measurements of the nanoparticle biodegradation, a mouse was fixed, and the area of the liver and spleen was scanned with the MPQ measuring coil. The maximum detected signal was used to assess the concentration of particles in the organ. Figure 2e shows that the amount of detectable magnetic material was decreasing with half-life t_1/2_ = 21 ± 4 days. Two months after the nanoparticle injection, only 7.4 ± 0.4% of the initial magnetic signal was detected in the liver and spleen. These data define the diagnostic window for the use of MPs as contrast agent for magnetic spectroscopic approaches, where the particles generated sufficient contrast against the liver background for 2 months and were well detectable in vivo.

### Evolution of nanoparticle biodistribution

**Fig. 3:**
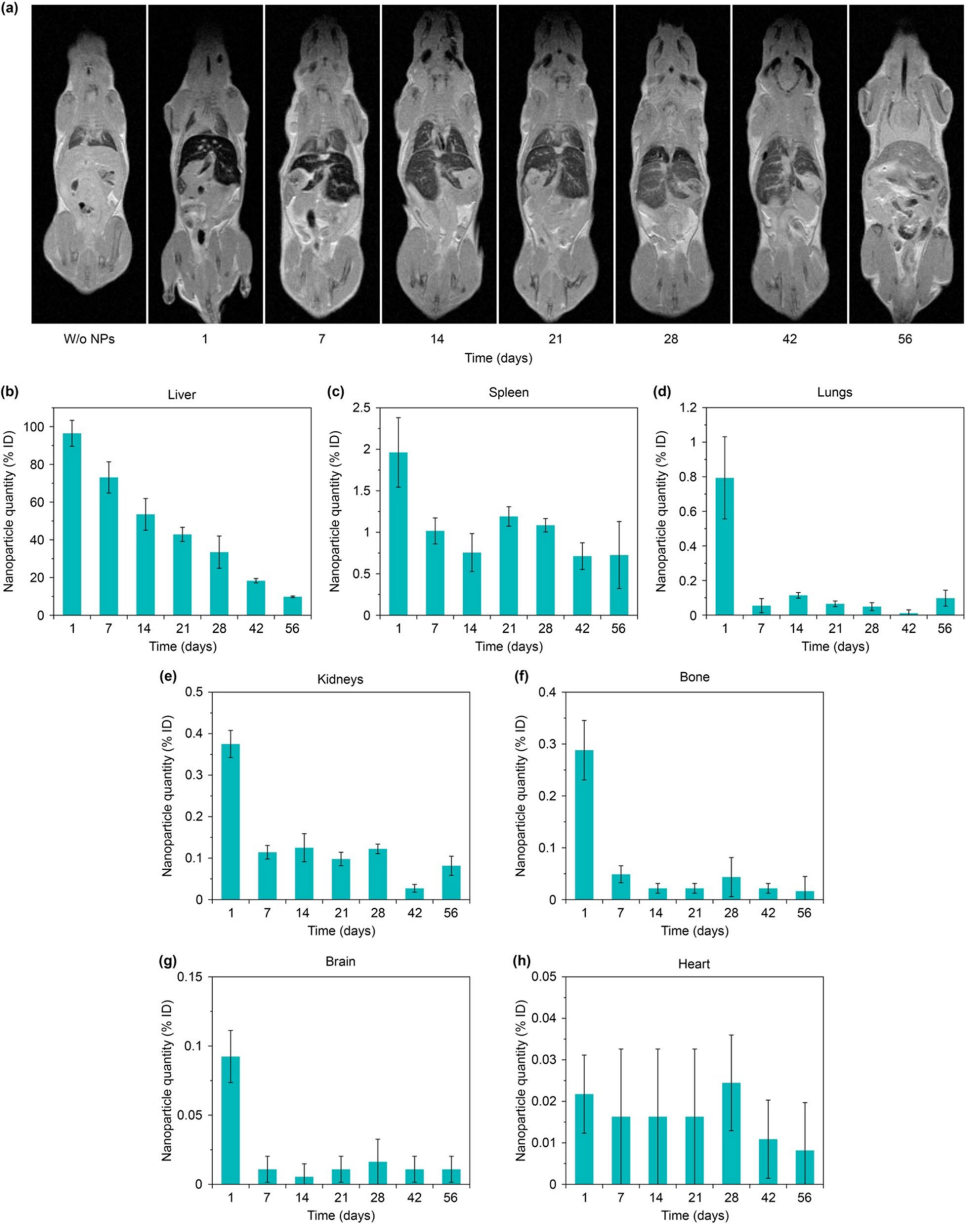
Investigation of nanoparticle biodistribution. **a** MRI data of 2-months evolution study of biodistribution in mice of FluidMAG-ARA nanoparticles. **b–h** MPQ data of 2-months evolution of MP concentration in different organs: liver (**b**), spleen (**c**), lungs (**d**), kidneys (**e**), bones (**f**), brain (**g**) and heart (**h**)

The observed drop of magnetic signal in the liver can be explained both by particle aging followed by their transition to weakly magnetic or linear magnetic forms of biogenic iron and by the redistribution of particles from the liver to other organs. Therefore, further we studied the evolution of nanoparticle biodistribution for 2 months after their administration (Fig. 3).

The ex vivo study of the nanoparticle biodistribution with the MPQ technique showed that one day after the injection, most of the nanoparticles (96 ± 6% ID) were localized in the liver (Fig. 3b). Then, they gradually degraded with time, and 3 weeks after the injection the magnetic signal in the organ decreased by more than 2 times, while after 8 weeks less than 10% of the initially injected dose was present in the liver. At the same time, a significant amount of the nanoparticles (2 ± 0.4% ID) was observed in the spleen (Fig. 3c). After 1 week, a magnetic signal from the nanoparticles in the organ reduced by half. However, for the next 2 months, the signal did not change significantly. That can be explained by active redistribution of the particles between the organs. The liver and spleen are the main organs of the mononuclear phagocyte system responsible for elimination of nanoparticles from the bloodstream. Nevertheless, 1 day after the particle administration, significant quantities of the nanoparticles (from 0.1 to 0.8% of the ID) were observed in lungs, kidneys, bones, brain, and heart (Fig. 3d–h). However, after 1 week, the magnetic signal from these organs significantly decreased and became almost undetectable by the MPQ technique. A similar redistribution of the particles between organs with nonspecific nanoparticle accumulation was previously described for other iron oxide nanoparticles [31].

### MRI contrast aging

Since the superparamagnetic nanoparticles are widely used as T2 contrasts for MRI, we investigated the evolution of their contrasting properties during their aging in the body (Fig. 3a). The best contrast was observed 1 day after the nanoparticle injection, the contours of the liver and spleen were clearly visible in the pictures. Besides, a slight contrast in comparison with untreated organs was observed in bones and kidneys (Additional file 1: Fig. S4).

Subsequently, during biodegradation of the particles in the body we observed a gradual decrease of the nanoparticle contrast properties. After 7 days, in all organs except liver and spleen the contrast returned to the control value, and the spleen contours were poorly observable. Quantitative contrast measurements showed that the MRI signal after 2 months of observation in the liver and spleen became 2 times lower than 1 day after the nanoparticle injection. Nevertheless, even after 8 weeks of the nanoparticle biodegradation, the liver contours were still clearly observable. Based on both our MPQ and MRI studies, we can declare that even 10% of the administered dose of FluidMAG-ARA particles (1.5 µg/g tissue) is sufficient to contrast the liver by magnetic techniques. Thus, intravenous injections of even small doses of magnetic nanoparticles can be used for long-term contrasting MRI studies of liver, but in all other organs the contrast properties of the nanoparticles quickly decay. It can be explained by uneven nanoparticle biodistribution between the organs (Fig. 3). Therefore, we assume that to contrast other organs except liver the nanoparticle redistribution or alternative administration methods should be used. Besides, the detected 2-month diagnostic window for the liver can be further used to evaluate the time-dependent contrast properties of existing MRI contrast agents based on magnetic nanoparticles and those under development.

### Evaluation of Fe-containing protein expression

**Fig. 4:**
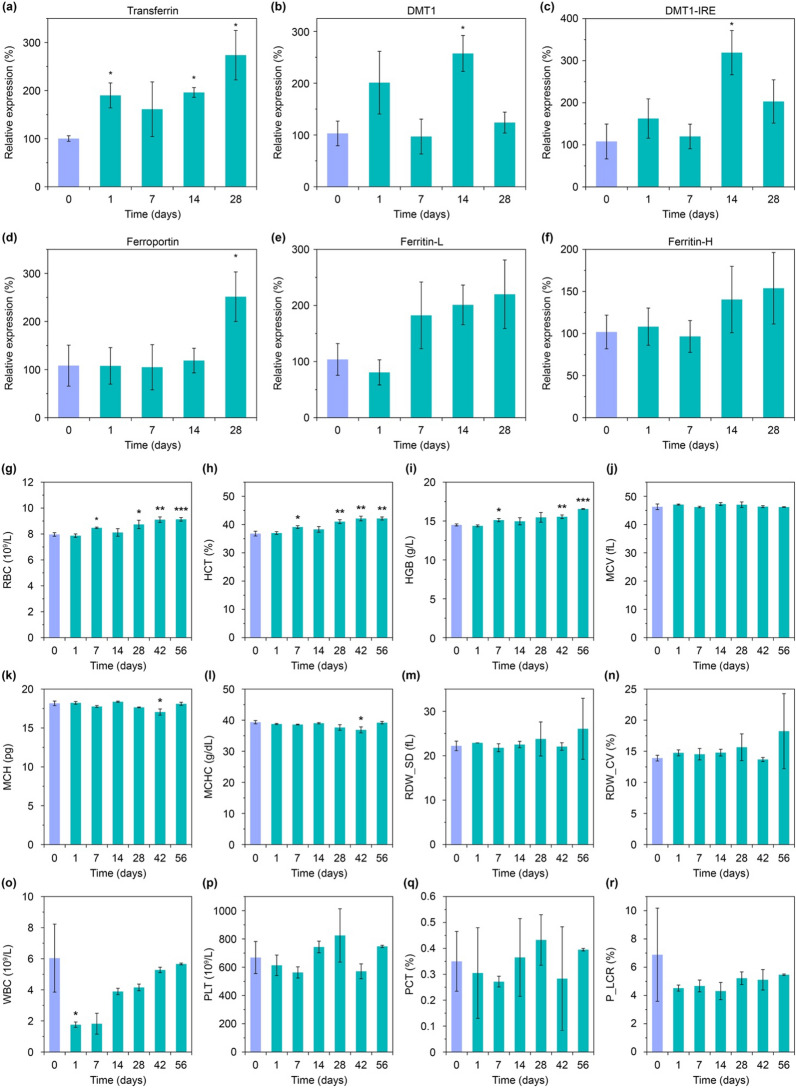
Gene expression and hematology analyses. **a**–**f** Expression levels of iron-containing proteins in the liver during 1 month after the nanoparticle administration: transferrin, DMT1, iron responsive element of DMT1 (DMT1-IRE), ferroportin, light and heavy chains of ferritin (Ferritin-L and Ferritin-H, respectively). **g**–**r** Hematological analysis within 2 months after the nanoparticle injection: red blood cell count (RBC), hematocrit (HCT), hemoglobin concentration (HGB), mean corpuscular volume (MCV), mean corpuscular hemoglobin (MCH), mean corpuscular hemoglobin concentration (MCHC), standard deviation of red cell distribution width (RDW_SD), coefficient of variation of red cell distribution width (RDW_CV), white blood cell count (WBC), platelet count (PLT), plateletcrit (PCT), platelet-large cell ratio (P_LCR). Asterisks indicate significant difference from the control group (violet bar in each graph): Welch’s t-test, *p < 0.05; **p < 0.01; ***p < 0.001

Iron is one of the most important metals in the body, involved in oxygen transport, DNA synthesis, oxidation-reductions, electron transport, etc. [32]. Its storage and transport are implemented by various proteins [33]. Therefore, the next goal of this study was to investigate the effect of iron release during MP biodegradation on expression of iron-containing proteins. Within 28 days after the particle injection, we studied the expression level in the liver of the main iron-containing proteins such as transferrin, DMT1 (with and without IRE domain), ferroportin, and ferritin (light and heavy chains) (Fig. 4a–f). It should be noted that the expression level of transferrin, which is mainly produced in the liver and is responsible for binding of Fe^3+^ ions, increased almost twice after 24 h since the nanoparticle administration and remained at high level after both 2 and 4 weeks (Fig. 4a). We assume that this can be explained by the high level of its involvement in the iron transport processes through the blood plasma [34], which redistribute biogenic iron between the organs during the nanoparticle biodegradation.

A similar expression change was also observed with the transport and storage of Fe^2+^ ions associated protein DMT1 (also known as Natural resistance-associated macrophage protein 2) (Fig. 4b, c). An expression of this protein also increased significantly and became more than 2.5 times higher in 2 weeks after the nanoparticle injection. However, for the next 14 days, the expression level decreased, and in 28 days post-injection it wasn’t statistically distinguished from the initial level. We hypothesize, this dependence can be explained by the high concentration of the released iron during the first 2 weeks of the nanoparticle degradation, but when the iron release rate drops, it may lead to the decrease in the expression level of DMT1 protein as a highly efficient iron carrier. We assume that the obtained tendency can indicate the reversibility of this process.

Besides, on the 28th day after the injection, we observed a significant increase in the expression level of intracellular iron exporter ferroportin (Fig. 4d). During the first 2 weeks, there were no noticeable changes in its expression level, but for the next 2 weeks it dramatically rose by more than 2.5 times. This increased protein amount can be involved in the processes of iron redistribution in the body, as well as in the processes of forming biogenic iron containing clusters for long-term iron storage.

**Fig. 5:**
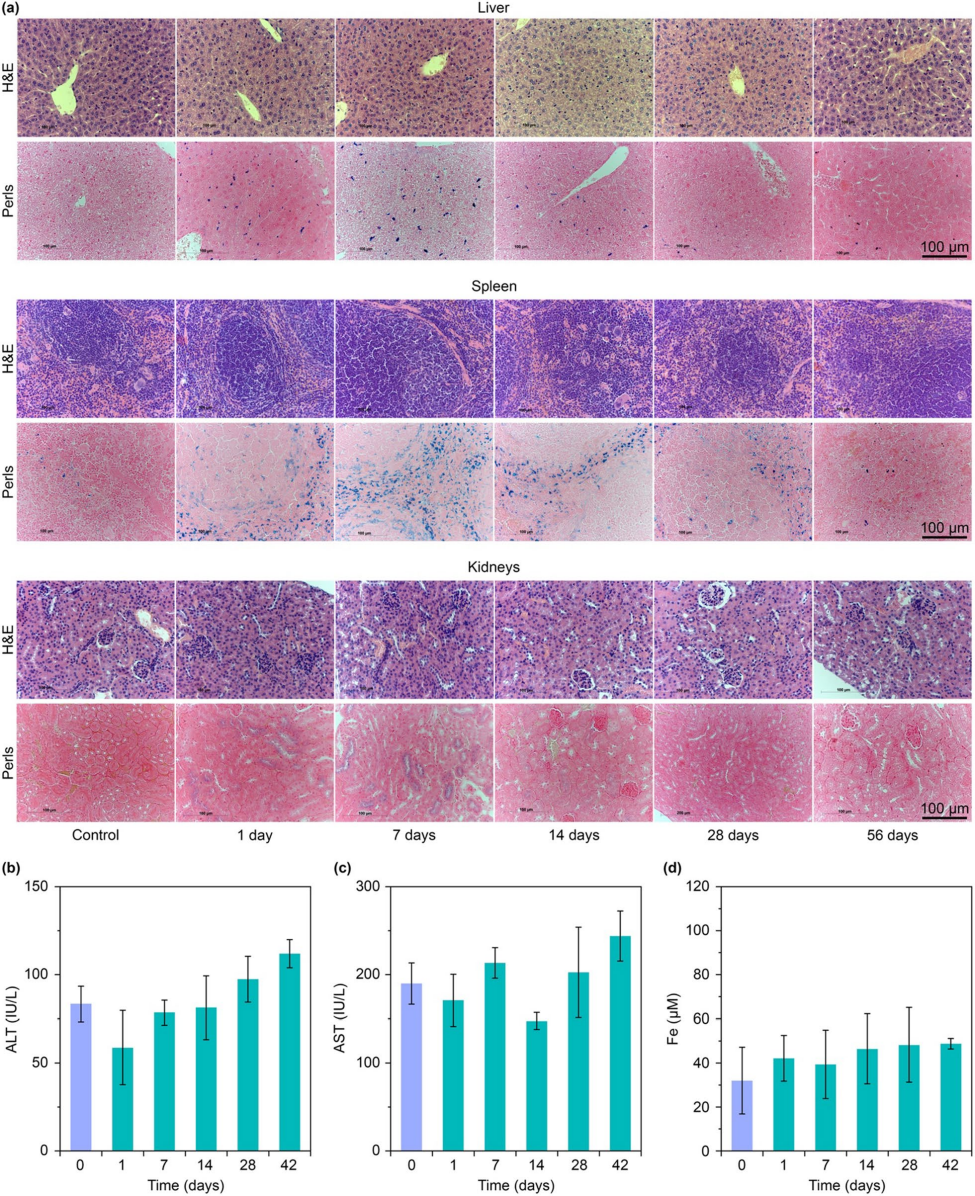
Histology and biochemistry. **a** Representative histological images of liver, spleen, and kidneys at various time points after the nanoparticle injection. Iron localization points appear as blue dots due to the Perls staining. **b**–**d** Biochemical parameters of murine plasma at various time points after the nanoparticle injection: **b** alanine aminotransferase activity (ALT), **c** aspartate aminotransferase activity (AST), **d** iron concentration (Fe). Violet bars in each graph indicate the control group (mice without the nanoparticle injection)

And finally, we investigated the expression level of ferritin (Fig. 4e, f) as one of the main proteins associated with intracellular iron-storage [13]. The level of its expression in the liver also tended to increase, however this increase was not statistically significant. We hypothesize, this small changes in the liver can be explained by the fact that the main iron storage organ is spleen, not the liver (Fig. 5a) [35].

Thus, biodegradation of the nanoparticles is accompanied by complex multi-stage processes of transporting and storing the released iron. During these processes, changes in expression levels can be observed for both proteins responsible for the iron transport outside the macrophages and for the proteins that implement iron transport between the organs through the blood.

### Hematological analysis

It has been previously shown that an increase in the iron amount in the body may affect blood formation processes [36]. Therefore, we investigated an impact of the nanoparticle biodegradation on hemopoiesis and thrombopoiesis. The hematological analysis was carried out for 56 days after the particle administration (Fig. 4g–r).

On 7, 28, 42, and 56 days after the nanoparticle injection, we observed 6–14% increase in the RBC amount and 2% increase of hematocrit (Fig. 4g, h). Those deviations are large but are still permissible within the normal non-pathological range [37]. A similar trend was also observed for hemoglobin ​​(46 increase at 7, 42, 60 days) (Fig. 4i). We assume that during the nanoparticle degradation, an increased iron release can stimulate erythropoiesis due to ferritin accumulation in macrophages, and that leads to the hemoglobin production in reticulocytes. From our data, we can suggest that the magnetic nanoparticles can be fast and, at the same time, a prolonged source of affordable iron and have pronounced erythropoietic activity.

It is also important to note that during the nanoparticle degradation, we did not observe any significant changes in the other erythrocyte parameters: mean corpuscular volume (MCV), mean corpuscular hemoglobin (MCH), mean corpuscular hemoglobin concentration (MCHC), and red cell distribution width (RDW) (Fig. 4j–n). These results may indicate that the nanoparticle degradation does not cause toxicity towards the RBC and do not disturb the hematopoiesis.

However, the nanoparticle intravenous administration led to 71% drop of WBC amount on the first day of the study (Fig. 4o). That can be explained by involvement of several WBC types such as lymphocytes, monocytes, and neutrophils in the nanoparticle recognition in blood with subsequent cell transport to mononuclear phagocyte system organs and cell destruction. Nonetheless, after complete removal of the nanoparticles from the bloodstream, the number of leukocytes gradually returned to the reference values. In addition, we didn’t observe any significant changes with platelet parameters such as platelet count (PLT), plateletcrit (PCT), and platelet-large cell ratio (P_LCR).

Thus, we can conclude that the nanoparticle injection can lead to a short-term reduction in the number of leukocytes, while the nanoparticle biodegradation can stimulate erythropoiesis with increasing the hemoglobin level. Besides both processes don’t affect thrombopoiesis.

### Histological assay

The next series of experiments was devoted to toxicological studies of nanoparticle degradation processes by the pathomorphological analysis of the liver, spleen and kidneys slices (Fig. 5a). In the liver, we didn’t observe any morphological changes on days 1, 7, 28, and 56 after the nanoparticle injection. However, on day 14 after the injection, the number of non-epithelial cells (Kupffer cells and lymphocytes) increased, and fine to average dystrophy was revealed. After Perls staining of the liver slices, we observed hemosiderin deposits on 1, 7, and 14 days after the nanoparticle injection. However, after 28 days, the number of iron-positive cells decreased, while after 56 days there were only extracellular small tight deposits of hemosiderin, observed probably due to the death of macrophages loaded with the nanoparticles.

In the spleen, on day 1 after the nanoparticle injection, we didn’t observe any morphological pathologies. However, after 7 days, a hyperplasia of white pulp and an increase of lymphoid nodules were detected. Then, on day 14 we identified wide light centers in the spleen, hyperplasia of periarteriolar lymphoid sheath (PALS) zones, and the increased quantity of megakaryocytes. However, on day 28, the light centers were not so wide compared to day 14, and the marginal zones were narrowing. Finally, on day 56, we observed an increase in the red pulp cellularity and the number of megakaryocytes. We hypothesized that all these morphological changes in the spleen could indicate an immunomodulatory or stress effect, which could be caused by the nanoparticle biodegradation. After Perls staining, we observed the hemosiderosis of the spleen during all 8 weeks of the experiment. On day 1 after the nanoparticle injection, hemosiderin deposits were detected in the red pulp and in the center of lymphoid nodules. On day 7, the number of hemosiderin deposits increased, but on day 14, their amount decreased in lymphoid nodules, and on days 28 and 56—in the entire spleen.

In the kidneys, on 1, 7, 14, and 56 days after the nanoparticle administration, there weren’t any morphological changes. However, on day 28, a dystrophic change in the epithelium of several tubules was revealed. After Perls staining on day 1 after the nanoparticle injection, we observed blue diffuse staining in the epithelium of some kidney tubules, which was mainly located in luminal area of the cells. On day 7, the intensity of diffuse staining was increased, but on day 14, it reduced, and on days 28 and 56 disappeared. We hypothesized that the presence of extra iron in the kidneys could be explained by non-specific accumulation of the smallest nanoparticles and their following degradation. This assumption well agrees with the obtained biodistribution data (Fig. 3e).

### Biochemistry

To study the organ specific toxicity of the nanoparticle decomposition products throughout the entire period of their degradation, we carried out a biochemical analysis of blood parameters such as alanine aminotransferase activity (ALT), aspartate aminotransferase activity (AST), and iron concentration (Fe) (Fig. 5b–d).

It is well known that ALT is an enzyme, which is mainly contained in liver and less—in kidneys, heart and skeletal muscles [38]. Almost any damage of the hepatic parenchyma contributes to the release of this enzyme into the blood. At the same time, AST enzyme is primarily located in tissues of intense metabolism (heart muscle, hepatic cells, kidneys, pancreas, and erythrocytes) [38]. Usually, after an acute or chronic damage of the tissues, these enzymes are released from cells. That leads to an increase of their activity in blood in direct dependence on the number of damaged cells. In our study of iron oxide degradation, ALT and AST activities remained within the normal ranges and were statistically not distinguishable from the control group values. Interestingly, the iron concentration remained at the level of the control group values ​​during the entire nanoparticle biodegradation process. We assume that the absence of changes in the iron concentration may be explained by the fact that the long-term nanoparticle degradation does not cause toxic iron poisoning. The slow release of iron from nanoparticles may contribute to its rational redistribution and deposition of its excess in the iron biogenic forms such as hemoglobin, ferritin, and hemosiderin. Thus, the results of blood biochemical analysis indirectly indicate that the long-term degradation of the injected nanoparticles does not cause any toxic effects in the internal organs and does not impact significantly the iron level in the blood plasma.

## Conclusion

In this paper, we studied the pharmacokinetics, biotransformation, and contrast properties of 40-nm superparamagnetic nanoparticles FluidMAG-ARA which could be important for the development of iron based theranostic agents. We have shown that magnetic nanoparticles with a core size of 20 nm almost fully degrade within 2 months. However, the biotransformation of such nanoparticles is a complex and multi-stage process, during which the nanoparticles can aggregate, change their magnetic properties, and redistribute between both cells and organs in the body. During the nanoparticle degradation, their iron-containing metabolites can circulate in the body, and the related iron release can change the expression level of proteins associated with iron transport and storage. We assume that the obtained data can be applicable to the theoretical assessment of biodegradation and biosafety of different magnetic nanoparticles, which are currently widely used in both clinic and scientific research.

## Supplementary Information

**Additional file 1: Figure S1.**Colloidal stability profile of nanoparticles in murine serum, PBS, and water. Size distribution by Intensity was used for analysis. **Figure S2.** (a–c) Logarithmic scale pharmacokinetics of FluidMAG-ARA nanoparticles in mice. The curves show kinetics of NP clearance from the bloodstream. Grey vertical lines indicate first 10 min of the fast elimination period. **Figure S3.** (a–c) Thermal dependence of the magnetization during the field-cooled (FC, top curve) and zero-field cooled (ZFC, bottom curve) measurements of liver samples (a) 1 day after FluidMAG-ARA NP injection, (b) 2 days after the NP injection and (c) the NPs in 1% agarose gel. TB – blocking temperature. **Figure S4.** MRI data of the 2 months evolution study of FluidMAG-ARA nanoparticles biodistribution in kidneys (top, green ellipses) and bones (buttom, blue ellipses).

## Data Availability

All data generated and analyzed during this research are included in this published article and additional file.
