# Supplementary material for: Influence of magnetic nanoparticle biotransformation on contrasting efficiency and iron metabolism
Source: J Nanobiotechnology. 2022 Dec 17;20:535. doi: 10.1186/s12951-022-01742-w (PMC9758463; doi:10.1186/s12951-022-01742-w)
Supplement: Supplementary file 1 — Additional file 1: Figure S1.Colloidal stability profile of nanoparticles in murine serum, PBS, and water. Size distribution by Intensity was used for analysis. Figure S2. (a–c) Logarithmic scale pharmacokinetics of FluidMAG-ARA nanoparticles in mice. The curves show kinetics of NP clearance from the bloodstream. Grey vertical lines indicate first 10 min of the fast elimination period. Figure S3. (a–c) Thermal dependence of the magnetization during the field-cooled (FC, top curve) and zero-field cooled (ZFC, bottom curve) measurements of liver samples (a) 1 day after FluidMAG-ARA NP injection, (b) 2 days after the NP injection and (c) the NPs in 1% agarose gel. TB – blocking temperature. Figure S4. MRI data of the 2 months evolution study of FluidMAG-ARA nanoparticles biodistribution in kidneys (top, green ellipses) and bones (buttom, blue ellipses). [file 12951_2022_1742_MOESM1_ESM.docx]

**Additional file**

**Influence of Magnetic Nanoparticle Biotransformation on Contrasting Efficiency and Iron Metabolism**

Alexey V. Yaremenko^#*^, Ivan V. Zelepukin^#^, Ilya N. Ivanov, Roman O. Melikov, Nadezhda A. Pechnikova, Dzhuliia Sh. Dzhalilova, Aziz B. Mirkasymov, Vera A. Bragina, Maxim P. Nikitin, Sergey M. Deyev & Petr I. Nikitin^*^

# - These authors contributed equally.

* - Corresponding authors’ e-mail: A.V. Yaremenko (aleksei.iaremenko@fulbrightmail.org), P.I. Nikitin (nikitin@kapella.gpi.ru)


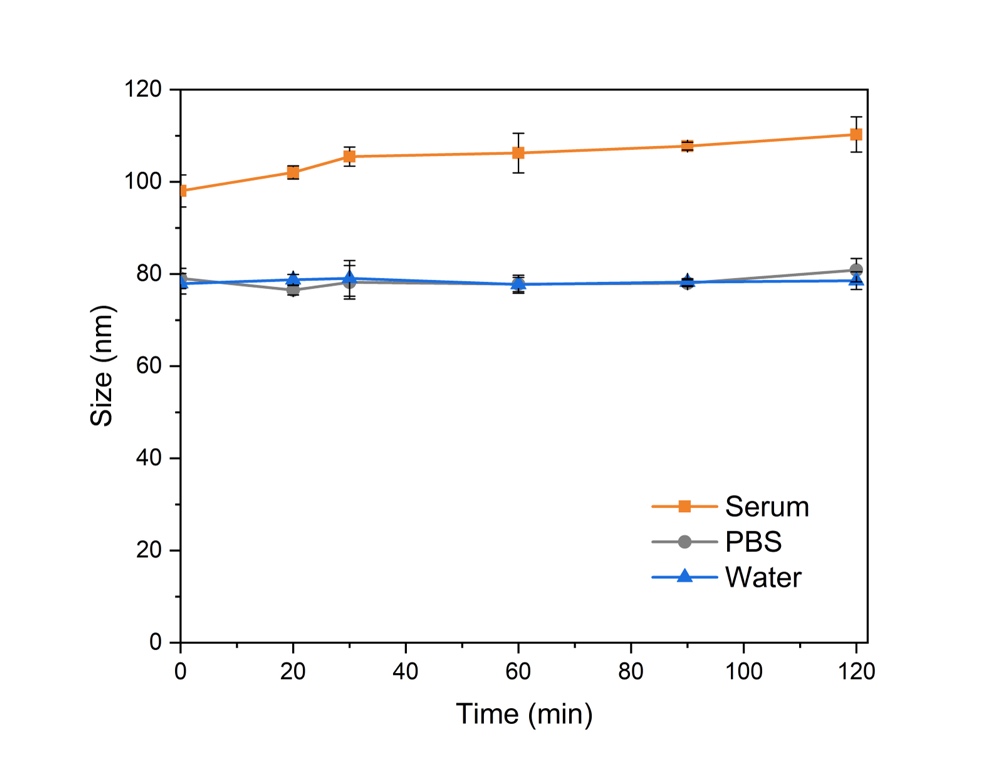


**Figure S1.** Colloidal stability profile of nanoparticles in murine serum, PBS, and water. Size distribution by Intensity was used for analysis.


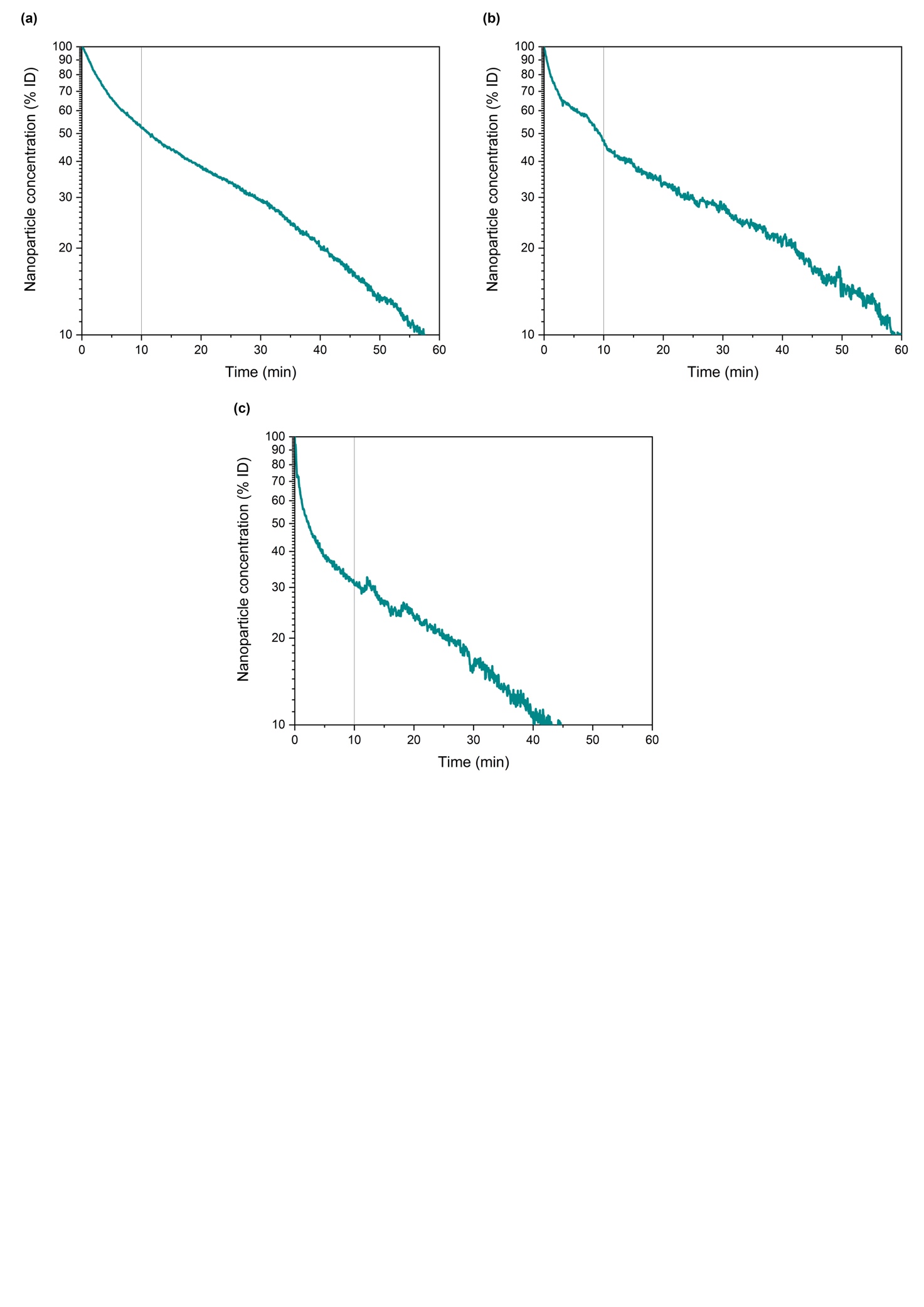


**Figure S2.** (a-c) Logarithmic scale pharmacokinetics of FluidMAG-ARA nanoparticles in mice. The curves show kinetics of NP clearance from the bloodstream. Grey vertical lines indicate first 10 min of the fast elimination period.

**
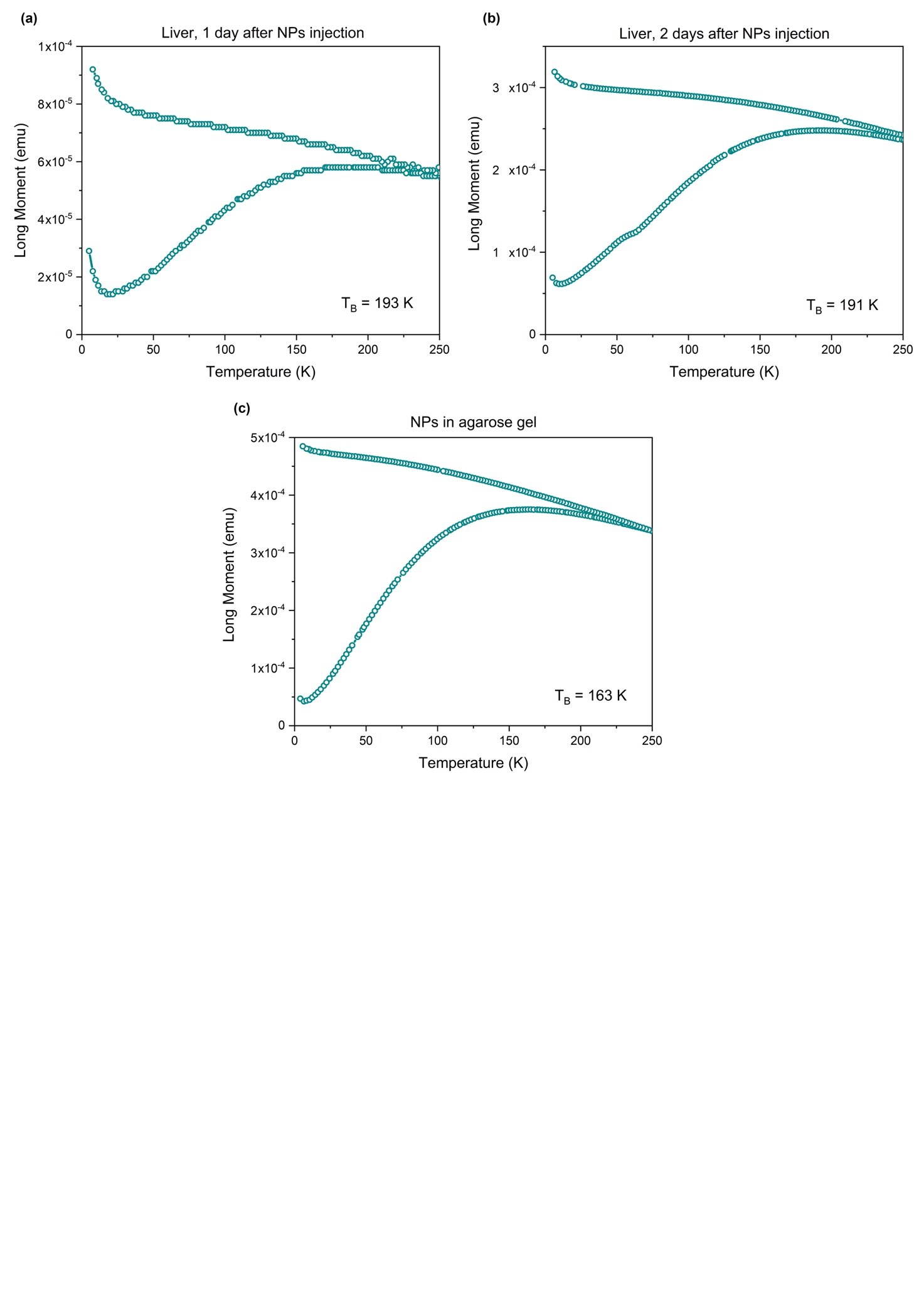
**

**Figure S3.** (a-c) Thermal dependence of the magnetization during the field-cooled (FC, top curve) and zero-field cooled (ZFC, bottom curve) measurements of liver samples (a) 1 day after FluidMAG-ARA NP injection, (b) 2 days after the NP injection and (c) the NPs in 1% agarose gel. T_B_ – blocking temperature.


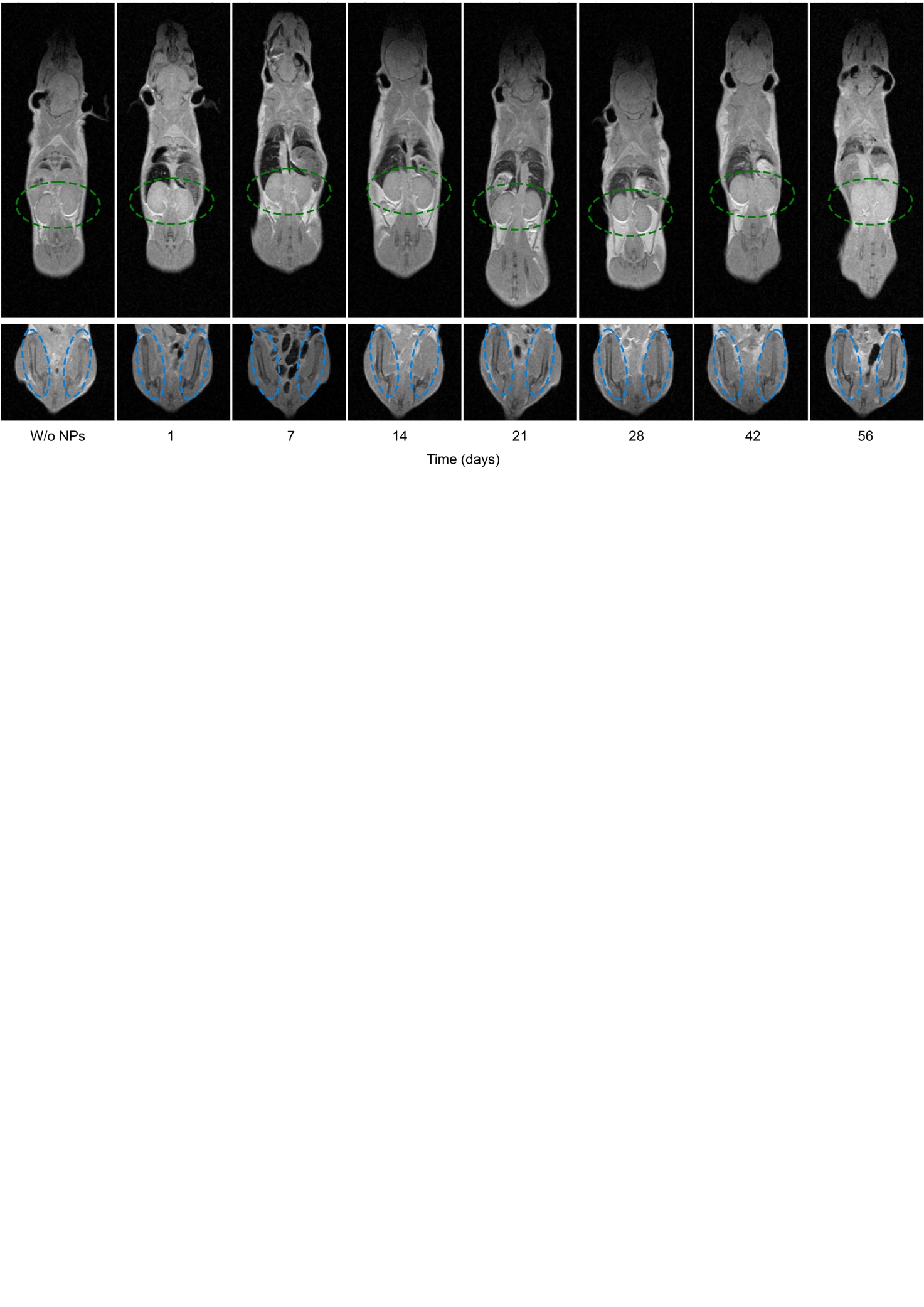


**Figure S4.** MRI data of the 2 months evolution study of FluidMAG-ARA nanoparticles biodistribution in kidneys (top, green ellipses) and bones (buttom, blue ellipses).
